# Proteinuria and Renal Dysfunction after Intravitreal Injection of Bevacizumab in Patients with Diabetic Nephropathy: A Prospective Observational Study

**DOI:** 10.22086/gmj.v0i0.1299

**Published:** 2018-10-16

**Authors:** Sina Bagheri, Banafshe Dormanesh, Mehrdad Afarid, Mohammad Mahdi Sagheb

**Affiliations:** ^1^Researcher, AJA University of Medical Sciences, Tehran, Iran; ^2^Shiraz Nephro-Urology Research Center, Shiraz University of Medical Sciences, Shiraz, Iran; ^3^Pediatric Department, Medical Faculty, AJA University of Medical Sciences, Tehran, Iran; ^4^Poostchi Ophthalmology Research Center and Department of Ophthalmology, Shiraz University of Medical Sciences, Shiraz, Iran

**Keywords:** Anti-VEGF, Bevacizumab, Diabetic Nephropathy, Diabetic Retinopathy, Glomerular Filtration Rate, Renal Dysfunction, Proteinuria

## Abstract

**Background:**

Proliferative diabetic retinopathy (PDR) is one of the most important microvascular complications among the patients with diabetes. Intravitreal anti-vascular endothelial growth factor (anti-VEGF) agent enacts a key role in PDR. Some studies have dealt with the systemic exposure to these agents after intravitreal administration. However, renal dysfunction following this therapy has scarcely been reported. Hence, this study aimed to determine the effect of intravitreal bevacizumab treatment on the deterioration of renal function and proteinuria.

**Materials and Methods:**

This present prospective observational study was performed on 40 patients with diabetic nephropathy and PDR and/or significant diabetic macular edema as the candidates for receiving intravitreal injection of bevacizumab. To evaluate renal function, changes in the urinary albumin-to-creatinine ratio (UACR), serum creatinine (SCr), and estimated glomerular filtration rate (eGFR) one month after injection were measured. Also, changes in systolic and diastolic blood pressures (BPs), plasma VEGF level, platelet, white blood cell (WBC) counts, and hemoglobin (Hb) level were measured at the baseline and one month after treatment.

**Results:**

The mean age of the patients was 60.3 ± 9.2 years, and 33 patients were female. The decrease in the plasma VEGF level and platelet count, as well as the increase in diastolic BP, and Hb level were significant. However, systolic BP and WBC count remained unchanged. There were no significant changes in UACR, SCr, and eGFR after the injection as compared to baseline (P>0.05).

**Conclusion:**

Our study indicated that intravitreal bevacizumab injection was not associated with renal dysfunction and proteinuria in patients with diabetic nephropathy. Nevertheless, diastolic BP and Hb level could increase after one month.

## Introduction

In recent years, some evidence has indicated the important role of vascular endothelial growth factor (VEGF) in the development of proliferative diabetic retinopathy (PDR) and diabetic macular edema (DME) [1]. Thus, intravitreal injection of anti-VEGF agents (e.g., bevacizumab) for the treatment of these two complications is increasing rapidly [2]. The intravenous administration of bevacizumab is used as an adjuvant therapy for many advanced cancers and has been associated with a significant reduction in the plasma VEGF level causing important adverse effects including hypertension, proteinuria, renal dysfunction,

and thromboembolism [3,4]. Although the intravitreal injection of bevacizumab is not approved by the Food and Drug Administration (FDA), it is widely used for the off-label treatment of PDR and DME. The intravitreal dose of bevacizumab is about 1/500th of the intravenous dose, and it is usually well-tolerated [5].

Nevertheless, side effects following anti-VEGF, such as elevated blood pressure (BP),

increased proteinuria, renal dysfunction, and thrombocytopenia, have been reported after the intravitreal injection of anti-VEGF drugs; however, limited data are available in this regard [6-11]. Like intravenous injection, intravitreal injection of bevacizumab is also absorbed systemically and decreases the plasma VEGF level to the significant amount of 17.1 pg/mL one month after injection [3,12]. Therefore, we investigate the effect of intravitreal injection of bevacizumab on the proteinuria and renal dysfunction of patients with diabetic nephropathy.

## Materials and Methods

### Patients

This single-center prospective observational study was carried out at Poostchi Ophthalmology Research Center at Shiraz University of Medical Sciences (Shiraz, Iran) from September 2017 to February 2018.

### Inclusion and Exclusion Criteria

Adult (>18 years of age) diabetic patients with proteinuria (urinary albumin-to-creatinine ratio [UACR] >30 mg/g in the spot urine) and PDR and/or clinically significant DME as the candidates for receiving intravitreal injection of bevacizumab were recruited and followed up for one month after the injection. Exclusion criteria included intravitreal injection of anti-VEGF drugs within four months prior to the study, previous vitrectomy, second injection requirement during one-month follow-up period, intravenous administration of anti-VEGF drugs within one year prior to the injection, end-stage renal disease (receiving hemodialysis or eGFR <15 mL/min/1.73 m^2^), pregnant or lactating women, any active form of cancers, and inflammatory or infectious disease.

### Treatment Protocol

Patients received 1.25 mg bevacizumab (Avastin, Roche, Basel, Switzerland) that was injected into the vitreous cavity 3.5 mm posterior to the limbus with a 30-gauge needle under sterile conditions in the operating room. After the injection, ciprofloxacin eye drops were administered to the patients four times per day.

### Data Collection

Proteinuria was measured by calculating UACR of the first-morning urine samples at baseline and one month after the injection. In order to reduce the variation in urinary albumin excretion, we instructed the patients to avoid vigorous exercise per day prior to overnight urine sampling. To evaluate renal function, serum creatinine (SCr), and estimated glomerular filtration rate (eGFR) (calculated by an equation developed by the Chronic Kidney Disease Epidemiology [CKD-EPI] Collaboration [13]) were measured at baseline and one month after the injection. Changes in the systolic and diastolic BPs, plasma VEGF level, platelet, WBC counts, and Hb level from baseline to one month after the injection were measured. At each visit (baseline and one month after the injection), BP of the patients were measured using the same automated BP monitor (Beurer BM-20, Beurer GmbH, Ulm, Germany) two times (with five-minute intervals) in a sitting position after a 15-minute rest, and the mean of two recorded BPs was reported. To measure VEGF level, blood samples were collected in tubes contained ethylenediaminetetraacetic acid (EDTA). Afterward, the samples were centrifuged, aliquoted, and stored at -70°C, and then analyzed in a single batch using Quantikine ELISA kit for human VEGF-D (R&D Systems, Minneapolis, Minnesota, USA) according to the manufacturer’s instructions.

### Ethical Statement

The Ethics Committee of Shiraz University of Medical Sciences reviewed and approved the study (code: 1396-01-18-15092), and then it was conducted in accordance with the Declaration of Helsinki Principles. All patients participating in the study signed the written informed consent form.

### Statistical Analysis

Statistical analysis was performed using SPSS Statistics, version 23.0 (SPSS, Inc., Chicago, IL, USA). Quantitative variables are reported as the mean ± standard deviation (SD) and number (percentage). We used Paired T-test and Wilcoxon Signed Rank Test to compare continuous variables. Correlations were calculated by Spearman rank correlation. A P-value less than 0.05 was considered statistically significant level.

## Results

Of 110 patients assessed for eligibility, 50 patients met the inclusion criteria. However, 10 patients did not attend the one-month follow-up period, and finally, 40 patients were enrolled in the final analysis. The mean age of the patients was 60.3 ± 9.2 years, and 33 (82.5%) patients were female. As shown in Table-1, the mean baseline plasma VEGF level significantly decreased from 345.3±239.6 pg/mL (range 86.1-1221.5) to 162.7±161.4 pg/mL (range 11.4-881.2) one month after injection (P<0.001). Nevertheless, there was no significant correlation between the decrease in plasma VEGF levels and changes in UACR (r = -0.01, P = 0.94). The mean UACR, eGFR, and SCr have not significant changes one month after injection as compared to baseline (P>0.05, Table-1). Although the change in mean systolic BP was not significant at one-month follow-up (95% confidence interval [CI]: -1‒3 mm Hg; P=0.21), the mean baseline diastolic BP significantly increased one month after injection (95% CI: 2‒8 mm Hg; P=0.002).

**Table 1:** Primary and Secondary Outcomes of Intravitreal Bevacizumab Injection at Baseline and One Month after Injection. Data Are Expressed as Mean ± SD

**Variables**	**Baseline**	**1 month**	**95% CI of difference**	**P-value**
**Plasma VEGF level (pg/mL)**	345.3 ± 239.6	162.7 ± 161.4	−227.5 to −137.7	<0.001*
**UACR (mg/g)**	372 ± 622	378 ± 605	−70 to 83	0.96
**SCr (mg/dL)**	**0.97** ± 0.27	0.98 ± 0.25	−0.03 to 0.05	0.63
**eGFR (mL/min/1.73 m^2^)**	71.3 ± 18.6	70.4 ± 18.6	−3.7 to 1.8	0.52
**Systolic BP (mm Hg)**	135 ± 12	136 ± 14	−1 to 3	0.21
**Diastolic BP (mm Hg)**	77 ± 10	82 ± 8	2 **to** 8	0.002*
**Platelet (cells/mm^3^)**	271250 ± 67699	244000 ± 70584	−42618 to −11882	<0.001*
**Hb (g/dL)**	12.38 ± 1.48	12.59 ± 1.43	0.01 to 0.42	0.04*
**WBC (cells/mm^3^)**	7485 ± 1746	7508 ± 1828	−334 to 379	0.90

**BP:** blood pressure, eGFR: estimated glomerular filtration rate, Hb: hemoglobin, SCr: serum creatinine, UACR: urinary albumin-to-creatinine ratio, VEGF: vascular endothelial growth factor, WBC: white blood cell, CI: confidence interval * Significant

As demonstrated in Table-1, the mean platelet counts and Hb level significantly decreased and increased one month after injection, respectively (P<0.001). However, the change in the mean WBC count was not significant.

## Discussion

In this prospective single-center observational study, we found that intravitreal injection of bevacizumab could not aggravate proteinuria or renal dysfunction in patients with diabetic nephropathy. However, it could decrease the plasma VEGF level and platelet count, and increase diastolic BP and Hb level. It has been documented that the systemic administration of VEGF-inhibitors increases the risk of developing proteinuria. Podocytes express VEGF that interacts with VEGF receptor-2 on glomerular capillary endothelial cells to keep normal renal function. Also, inhibition of VEGF leads to loss of endothelial fenestrations and proteinuria [4,14]. There are few cases of proteinuria following intravitreal injection of VEGF-inhibitors in the literature [6,8,10].

Although the plasma VEGF levels decreased significantly one month after the intravitreal injection of bevacizumab in our patients, consequently proteinuria was not increased. Similar to our results, Glassman et al. [15] concluded that UACR change was not markedly one year after intravitreal injection of VEGF-inhibitors. The differences in the development of proteinuria following the systemic versus intravitreal administration of bevacizumab might be explained by lower doses used, lower half-life, localized route of injection, and formulations. An uncommon adverse effect following the systemic anti-VEGF therapy is renal dysfunction [4]. Some evidence revealed that acute renal dysfunction could occur after intravitreal administration of VEGF-inhibitors [7,10,11]. Nevertheless, intravitreal injection of bevacizumab failed to change the renal function in our study. In line with our study, Kameda et al. [16] reported that eGFR was not changed after intravitreal injection of anti-VEGF drugs in patients with diabetes and chronic kidney disease. Hypertension is another adverse event associated with the systemic anti-VEGF treatment which occurs in 11-43% of the patients. VEGF increases the production of nitric oxide (a potent vasodilator) through promoting the transcription of endothelial nitric oxide synthase. Thus, inhibition of VEGF leads to the decreased production of nitric oxide and subsequent hypertension [17]. In our study, even though mean systolic BP was not changed one month after intravitreal injection of bevacizumab, mean diastolic BP increased by five mm Hg. In another study, the mean systolic BP increased by 17 mm Hg three months after the intravitreal administration of bevacizumab, while the mean diastolic BP remained unchanged [18]. In contrast, Glassman et al. [15] showed that intravitreal injection of anti-VEGF drugs was not able to increase the risk of developing or worsening hypertension over two years of follow-up. It is reported that the systemic use of bevacizumab increases the risk of neutropenia and thrombocytopenia, while it lowers the risk of anemia [19]. VEGF receptor-2 is expressed on early hematopoietic precursors, including hemangioblast and stem cells [20,21], and an early defect in the development of hematopoietic cells was observed after receptor blockade in mice models [22,23]. Moreover, it seems that VEGF inhibition can increase erythropoietin production and erythrocytosis since VEGF has a negative regulatory role in hepatic erythropoietin synthesis [24]. Severe immune-mediated thrombocytopenia after intravitreal bevacizumab injection has been reported [9]. Intravitreal administration of bevacizumab decreased the mean platelet count in our study and resulted in new-onset thrombocytopenia in three (7.5%) patients and aggravated pre-existing thrombocytopenia in one (2.5%) patient. It also slightly increased the mean Hb level while it showed no change in the mean WBC count. The above-mentioned studies investigated the effect of intravitreal VEGF-inhibitors injection on proteinuria and renal dysfunction was both retrospectives [15,16]. While, to the best of our knowledge, this study is the first prospective research that evaluates under-scrutiny complications occurring after the intravitreal administration of anti-VEGF agents. In contrast to previous studies, our study was performed on diabetic patients with pre-existing proteinuria and showed that intravitreal bevacizumab injection was relatively safe in this high-risk group of patients. Even though bevacizumab did not increase the mean UACR in our study, it is important to mention that UACR increased in 18 (45.0%) patients (range: +3 to +894 mg/g).

Hence, monitoring of proteinuria should be recommended in patients receiving intravitreal anti-VEGF agents. Our study has some limitations. First, our sample size was relatively small. Second, we merely followed-up the patients for one month after injection and failed to measure the UACR or SCr at longer intervals. Finally, we measured BP just at baseline and post-injection visit; therefore, changes resulted from antihypertensive medications that are likely to affect our findings were overlooked. Hence, future cohort studies and clinical trials with long follow-up and a larger sample size are recommended.

## Conclusions

Our study suggests that intravitreal injection of bevacizumab could not change UACR or renal function among patients with diabetic nephropathy. Also, such a therapy should be cautiously used in patients with thrombocytopenia or hypertension.

## Conflict of Interest

The authors declare that there is no conflict of interest regarding the publication of this article.
